# Development and optimization of an immunoassay for the detection of Hg(II) in lake water

**DOI:** 10.1002/fsn3.991

**Published:** 2019-04-06

**Authors:** Xuexue Jin, Rong He, Xingrong Ju, Jie Zhang, Mingjie Wang, Changrui Xing, Jian Yuan

**Affiliations:** ^1^ College of Food Science and Engineering/Collaborative Innovation Center for Modern Grain Circulation and Safety/Key Laboratory of Grains and Oils Quality Control and Processing Nanjing University of Finance and Economics Nanjing China; ^2^ School of Grain Science and Technology JiangSu University of Science and Technology Zhenjiang China

**Keywords:** antibody, Hg(II), immunoassay, lake water, tap water

## Abstract

In this paper, an indirect competitive enzyme‐linked immunosorbent assay (IC‐ELISA) has been developed and optimized to detect Hg(II) in tap water and lake water based on a monoclonal antibody (mAb‐A24). Some stabilizing additives (Gelatin, bovine serum albumin [BSA], polyvinyl alcohol [PVA], and polyvinyl pyrrolidone [PVP]) and surfactant (Tween‐20) have been investigated thoroughly in the optimization process. Under the optimal condition, the 50% half maximal inhibitory concentration (IC_50_) and limit of detection (LOD) were 1.68 and 0.079 ng/ml, respectively. These anti‐Hg mAbs also have some affinity with methyl mercury (CH_3_Hg) and with no cross‐reactivity with other thirteen metal ions. The developed method has shown satisfactory recovery of Hg(II), ranged between 91% and 116%, from tap water and lake water. Therefore, this immunoassay can be used to detect trace Hg(II) in environment water.

## 1 INTRODUCTION

Heavy metal contamination is still one of the most severe global environmental problems as the result of enrichment processes (Zhang et al., 2018). It is noteworthy that mercury pollution has ever been a breaking event, known as “Minamata disease,” caused by the methylmercury (CH_3_Hg) poisoning (Harada, 1995). Mercury is ubiquitous in the biosphere including air, soil, water, and food, and its main emission resources are fossil fuel burning, solid waste combustion, and chemical manufacturing (Cheng & Hu, 2012). Being contrary to organic degradation, mercury is not biodegradable and can be bio‐accumulated along the food chain. Hg(II) is a solvated ion with high cellular toxicity and carcinogenic potential and becomes stronger toxicity speciation through microbial mercury methylation even in polar marine environments (Gionfriddo et al., 2016). These organic mercury speciations contain methylmercury, ethylmercury, and phenylmercury. Typical symptoms of methylmercury poisoning are sensory disturbances, dysarthria, and ataxia with the damage in central nervous system or digestive systems (Korbas et al., 2010). Mercury is defined as “priority hazardous substances” by the Agency for Toxic Substances and Disease Registry. The established maximum residue limits (MRLs) for total Hg(II) in drinking water and for methyl mercury in carnivorous fishes are 1 ng/ml and 1.0 mg/kg, respectively.

Some typical analytical methods including cold vapor atomic fluorescence spectrometry (CV‐AFS; Bagheri & Gholami, 2001), electrothermal vaporization‐inductively coupled plasma‐mass spectrometry (ETV‐ICP‐MS; Vanhaecke, Resano, & Moens, 2002), and a high performance liquid chromatography system (HPLC) coupled to an inductively coupled plasma‐mass spectrometer (ICP‐MS; Wang et al., 2007) are most commonly used for the detection of mercury and methylmercury. These conventional analytical methods are accurate and sensitive. However, they require expensive instrument, technical staff, and complicated sample treatments, and are not portable for *on‐site* detection. Recently, antibody‐based immunoassays have been used as an interesting alternative for the detection of lead (Pb(II)) (Khosraviani et al., 2000; Kuang et al., 2013; Sun et al., 2018), cadmium(Cd(II)) (Xing, Kuang et al., 2014; Xu et al., 2016), copper(Cu(II)) (Xing, Hao, Hao, Liu, Xu, & Kuang, 2014), mercury(Hg(II)) (Wang et al., 2012; Zou, Cui, Liu, Song, & Kuang, 2017), and indium(In(III)) (Boden, Colin, Barbet, Doussal, & Vijayalakshmi, 1995). These antibodies could recognize metal chelates, such as ethylenediaminetetraacetic acid (EDTA) metal chelate, diethylenetriaminepentaacetic acid (DTPA) metal chelate, and isothiocyanobenzyl‐EDTA (ITCBE) metal chelate. In our previous work, Hg‐ITCBE‐Keyhole limpet hemocyanin (Hg‐ITCBE‐KLH) has been used successfully to produce monoclonal antibodies for recognition of mercury‐EDTA conjugates. Till now, mercury antibodies have been reported against EDTA‐Hg or Hg^2+^ induced by the Hg‐ITCBE‐KLH (Xing, Liu, Liu, Zhang, Kuang, & Xu, 2014), Hg‐MNA‐BSA (Wang et al., 2012; Zou et al., 2017), or Hg‐GSH‐KLH (Wylie et al., 1992). However, more inspection of antibody‐based ELISA component and application for the detection of Hg(II) are still needed.

In this paper, we have developed a fast and simple enzyme‐linked immunosorbent assay (ELISA) based on our newly screened monoclonal antibody (mAb) for the detection of Hg(II). Antibody sensitivity and specificity were measured before the establishment of immunosorbent assays. Effects of the protection reagents, pH and surfactant on antibody sensitivity have been evaluated. Samples spiked in tap water and lake water were tested by the developed method to evaluate the accuracy.

## 2 MATERIALS AND METHODS

### 2.1 Chemicals and materials

Metal ions’ standards, including Hg(II), CH_3_Hg, Cr(III), Cr(VI), Mg(II), Cu(II), Ni(II), Fe(II), Co(II), Mn(II), Zn(II), Au(II), As(III), Ca(III), and Cd(II), were purchased from the National Standard Material Center (National Institute of Metrology, Beijing, China). 6‐mercaptonicotinic acid (MNA) was purchased from Sigma‐Aldrich (Shanghai, China). All chemicals were ultrapure grade.

### 2.2 Synthesis of immunogen and coating antigen

Hg was conjugated with BSA through 6‐mercaptonicotinic acid (MNA) by EDC/NHS method as described previously (Liu, Xing, Yan, Kuang, & Xu, 2014). Hg‐MNA‐BSA could expose the Hg alone to the immune system. Briefly, 15 mg MNA, 16 mg EDC, and 23 mg NHS were dissolved in 0.5 ml DMF and stirred overnight at room temperature. Then, the solution was centrifuged at 20,000 *g* for 10 min, and the supernatant was added dropwise to 40 mg BSA solution (dissolved in 3 ml of 0.1 M carbonate buffer saline, pH 9.0). The mixture was kept reacted for 12 hr and dialyzed 2 days in 0.01 M phosphate‐buffered saline (PBS), pH 7.4. Next, Hg was added dropwise to the dialyzed protein solution under pH 8 by 0.1 M potassium hydroxide (KOH). The mole ratio of Hg to MNA was 1:1.3. The reaction was incubated at room temperature overnight. Finally, the solution was centrifuged at 2,800 *g* for 30 min by ultrafiltration and dissolved in 0.01 M PBS, pH 7.4. The centrifugation process was repeated three times. The final concentration of immunogen was 2 mg/ml. Hg‐MNA‐Ovalbumin (Hg‐MNA‐OVA) was synthesized as the same process and used as coating antigen. Both Hg‐MNA‐BSA and Hg‐MNA‐OVA were stored at −20°C for further use.

### 2.3 Hg concentration detection by ICP‐MS

Before the immunization, the amount of Hg(II) in the immunogens and coating antigen was detected by ICP‐MS. 0.2 ml samples were digested by concentrated HNO_3_ and diluted by 2% HNO_3_ to 10 ml. Hg standard solutions (0, 0.1, 0.2, 0.5, 1, 2, and 5 ng/ml) were also prepared as the standard curve and detected.

### 2.4 Immunization and mice screening by ELISA

The BALB/c mice, 6–8 weeks of age, were chosen and immunized five times using immunogen of Hg‐MNA‐BSA, at an interval of 28 days (Xing, Liu et al., 2014). The immunization doses were 100, 100, 100, 50, and 50 µg, respectively. After fifth immunization, the mice show the highest affinity and specificity to Hg and no cross‐reactivity to MNA and other metal ion was selected for cell fusion. The selected mice were given final booster injection intraperitoneally (30 μg of immunogen directly dissolved in 200 μl of physiological saline). After 3 days, the mice were sacrificed and the spleen was taken to cell fusion according to the previously described steps.

### 2.5 Preparation of monoclonal antibodies

The cell fusion experiment was performed by conventional PEG fusion technology to obtain monoclonal antibodies. At the seventh day after cell fusion, the supernatant secreted by the hybridoma cells was screened by ELISA to find the positive cells. Positive cells were subcloned three times by limited dilution method. The sensitivity, subtypes, and cross‐reactivity of antibody purified from ascites fluid were detected.

### 2.6 Competitive indirect ELISA (IC‐ELISA)

Conventional IC‐ELISA was carried out according to previous research. Hg‐MNA‐OVA (0.1, 0.2, 0.3, 0.5, 1, and 2 µg/ml) was diluted in CBS (0.01 M pH 9.0). 100 µl diluted coat antigen was added to the well of microtitre plates and incubated for 2 hr at 37°C. The plates were washed three times using PBST and blocked by the CBS containing 0.1% gelatin at 4°C overnight. After washing the plates twice, those plates were dried for 20 min and preserved at 4°C for use. The color development was in conformance with the standard process.

Based on a noncompetitive two‐dimensional titration, OD values around 1.8 were selected for sensitivity detection. A series of Hg standard solution prepared in pH 7.4 PBS was tested according to the previously described steps. The standard curves under different combinations were obtained and IC_50_ was calculated. Concentrations of coating conjugates and concentrations of antibody provided the inhibition curve with the lowest IC_50_ were selected for further assay development.

### 2.7 Assay optimization

The effects of pH, blocking solution, and surfactant on the competitive assay were further optimized.

#### 2.7.1 pH effect

To evaluate the effect of the pH of sample solution on the sensitivity, PBS buffers with pH 3–8 were prepared and used to dilute Hg standard solution. All other assay processes were the same as described before. All the experiments were performed three times.

#### 2.7.2 Blocking and antibody dilution buffer

The type and concentration of blocking reagents are one of the important factors to determine the stability and validity of ELISA. In this paper, Hg was recognized by antibody alone without the help of Hg chelate compound. Protein or other polymer in the blocking and antibody dilution buffer may interfere the interaction of Hg and antibody. Gelatin (0.1%, 0.2%, and 0.5%), BSA (0.1%, 0.2%, and 0.5%), PVA (0.05%, 0.1%, and 0.2%), and PVP (0.05%, 0.1%, and 0.2%) were prepared as the blocking solution and tested. Gelatin (0.05%, 0.02%, and 0.01%), BSA (0.05%, 0.02%, and 0.01%), PVA (0.05%, 0.02%, and 0.01%), and PVP (0.05%, 0.02%, and 0.01%) were prepared as the antibody dilution buffer and tested. Tween‐20 (0.50%, 0.20%, 0.10%, 0.05%, 0.02%, and 0.01%) in antibody dilution buffer were also prepared and tested.

### 2.8 Cross‐reactivities (CRs)

Other metals such as CH_3_Hg, Cr(III), Cr(VI), Mg(II), Cu(II), Ni(II), Fe(II), Co(II), Mn(II), Zn(II), Au(II), As(III), Ca(III), and Cd(II) were tested by the established IC‐ELISA. Metal ions with different concentration range between 1 and 1,000 ng/ml were prepared in PBS and tested. The CR was calculated based on the following formula.

$$\text{CR}\left(\%\right)=\left({\text{IC}}_{50}\;\text{of Hg}\left(\text{II}\right)/{\text{IC}}_{50}\;\text{of other tested metals}\right)$$

### 2.9 Fortification of Hg(II) in tap water and lake water

A standard dilution series of Hg(II) was spiked into tap water and lake water with concentration of 1, 2, and 5 ng/ml. The tap water was obtained in the laboratory, and the lake water was sampled in Xuanwu Lake in Nanjing. Then, the samples were conditioned by the addition of a 10% volume of 0.1 M PBS buffer with final pH 6. Then, the samples were tested by IC‐ELISA and compared with that obtained by ICP‐MS.

## 3 RESULTS AND DISCUSSION

### 3.1 Characterization of immunogen and coat antigen

It is well know that hapten alone could not induce immune response. The hapten should be coupled with macromolecular protein, such as BSA, KLH, or OVA, to form antigen. Through a bifunctional coupling reagent, Hg(II) could conjugate with BSA to form an immunogen, exposing Hg(II) to the immune system. In our previous research, ITCBE has been shown universal ability to couple Pb(II), Cd(II), Hg(II), Cu(II), and Cr(III) to KLH and produce antibodies to their corresponding EDTA or ITCBE chelate. In this paper, MNA has been chosen as a bifunctional coupling reagent. The carboxyl group could react with amino group on protein, bringing in a mono‐thiol group which could combine with Hg(II). The antibody produced by this method could recognize Hg(II) without the help of MNA.

**Figure 1:**
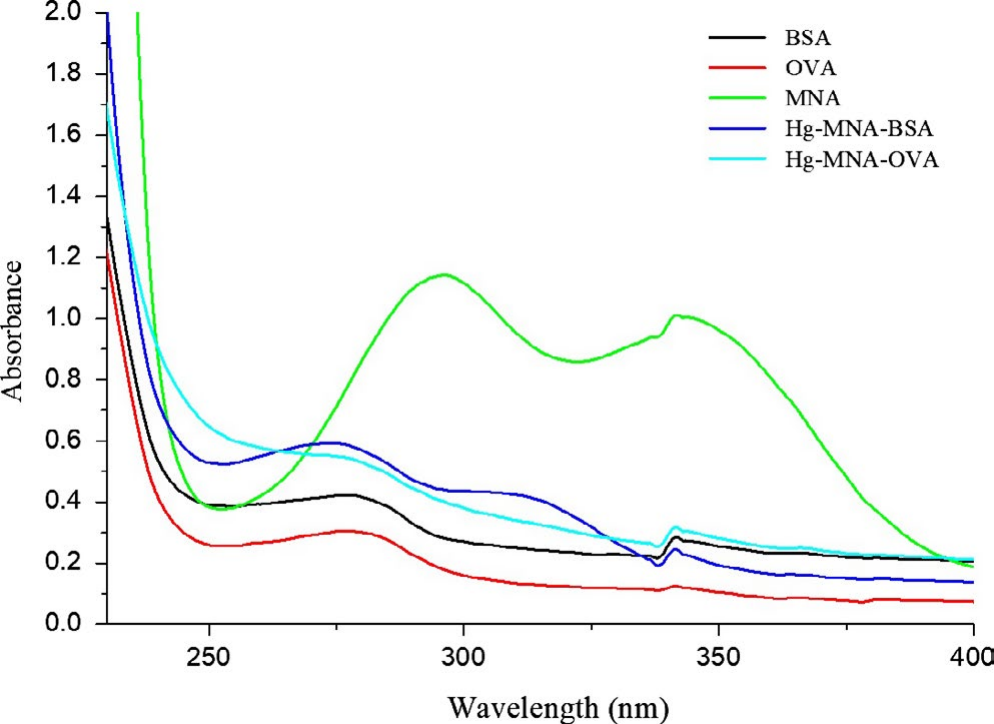
The UV spectrum of OVA, BSA, MNA, Hg‐MNA‐BSA, and Hg‐MNA‐OVA

Therefore, the synthesized antigens were characterized by ICP‐MS and UV‐visible spectrophotometer. The amount of Hg(II) was assured by ICP‐MS and the molar coupling ratio (Hg(II) to BSA) is 13:1, indicating Hg(II) has been successfully linked to the protein. The UV scanning of Hg‐MNA‐BSA and Hg‐MNA‐OVA is shown in Figure 1. The characteristic peaks of BSA and OVA were located at 280 nm. The characteristic peaks of MNA were located at 300 and 350 nm. The conjugate of Hg‐MNA‐BSA and Hg‐MNA‐OVA both had the characteristic peaks located at 280 and 320 nm, indicating successfully conjugation of MNA. These identifications indicated that Hg(II) has been successfully coupled with BSA and OVA and used in the mice immunization (Wang et al., 2012; Zou et al., 2017).

### 3.2 Characterization of antisera

After the five injections of Hg‐MNA‐BSA, antisera were collected from the tail of each mouse and tested. Table 1 shows the reactivity of mouse 4 tested by the IC‐ELISA. These antisera show best sensitivity when 100 ng/ml Hg(II) was tested. We also evaluate the effect of chelating reagent to the antibody interaction. The results showed 10 mM MNA will obviously affect the antibody recognition of Hg(II). 1 mM EDTA will form Hg‐EDTA and show decreased affinity between the Hg‐EDTA and antibody. This result demonstrated that reactivity of antibodies with Hg(II) was not dependent upon the presence of MNA and inhibited by high concentration of MNA and EDTA.

**Table 1:** Sensitivity detection of mouse antisera with different chelate

Hg(II) concentration	PBS containing different chelate				
	PBS Only	10 mM MNA	1 mM EDTA	0 mM MNA	10 mM MNA
0 ng/ml Hg	2.5065	0.9637	2.0488	2.3804	0.8184
100 ng/ml Hg	0.5691	0.5466	1.4843		

As CH_3_Hg was the organic form of Hg(II), CH_3_Hg has been tested for their possible recognition by antibody shown in Table 2. Furthermore, we also synthesized CH_3_Hg‐MNA‐OVA as the heterologous coating and used to test their sensitivity to Hg(II) and CH_3_Hg. Although both of them could be identified, the results indicated that antibody molecules containing an epitope with higher affinity to Hg(II). Finally, mouse 4 demonstrated the highest sensitivity to Hg(II) and used for cell fusion.

**Table 2:** IC_50_ of antisera tested by homologous coating (Hg‐MNA‐OVA) and heterologous coating (CH_3_Hg‐MNA‐OVA)

Coating type	Standard solution containing metal species	IC_50_ (ng/ml)
Hg‐MNA‐OVA	Hg(II)	10
	CH_3_Hg	20
CH_3_Hg‐MNA‐OVA	Hg(II)	20
	CH_3_Hg	50

### 3.3 Characterization of mAb and assay optimization

After cell fusion, subclone, and purification, mAb A24 with highest titer and sensitivity was obtained. The isotypes of obtained mAbs were IgG1 type. Before the practical application of this antibody for the detection of environmental water samples and crop, it is essential to optimize the sensitivity of ELISA and stability of kit components (Kolosova, Shim, Yang, Eremin, & Chung, 2006). Therefore, some additives were tested for their stabilizing effect and interference on antigen immobilized and antigen‐antibody interaction in the microwell plates. In order to protect of immobilized antigen, sugars (sucrose), polymers (PVP, PVA, PEG, etc), gelatin, or BSA were added during the drying process and storage (Dankwardt, Müller, & Hock, 1998).

First, pH of coating solution was investigated. Electrostatic interaction plays an important role in the efficiency of antigen immobilization on the surface of binding sites of the polystyrene. Commonly, carbonate (pH 9.6), Tris‐HCl (pH 8.5), and PBS (pH 7.2) were all suitable for BSA or OVA immobilization on the polystyrene surface by passive adsorption. In the presence of detergents or other additives such as PVA and BSA, the binding sites of the polystyrene surface are occupied and antigen immobilization prevented (Gardas & Lewartowska, 1988). These additives, such as gelatin or BSA, are usually used in blocking solution and the antibody dilution solution for the prevention of antibody degradation and non‐specific adsorption. However, Hg(II) easily bound with BSA showing binding constants with 1:1 stoichiometry of K_BSA/Hg_ = (5.47 ± 0.70) × 10^3^ M^−1^ by fluorometric titration measurements (Ma et al., 2008). Here, different concentration of gelatin were used in the blocking solution and antibody dilution solution to survey their effect to the sensitivity. As shown in Table 3, this interference also existed in high gelatin concentration containing dilution solution. When the concentration of gelatin was more than 0.05%, the sensitivity of ELISA was decreased 10‐fold as Hg(II) in the standard solution absorbed. There is no big difference between PBS and CBS used as the coating buffer. We chose CBS in the following experiment. The interference of gelatin in antibody dilution solution for Hg(II) detection indicated more investigation is needed.

**Table 3:** Optimization of coating solution, blocking solution, and gelatin concentration in antibody dilution solution

Coating solution/Blocking solution	Gelatin concentration in antibody dilution solution (%)	OD of blank	IC_50_ (ng/ml)
0.01 M PBS/0.01 M PBS containing 0.2% gelatin	0.02	2.287	5
	0.05	2.260	5
	0.10	2.161	50
0.01 M PBS/0.01 M CBS containing 0.2% gelatin	0.02	1.964	5
	0.05	1.879	5
	0.10	2.128	50
0.01 M CBS/0.01 M PBS containing 0.2% gelatin	0.02	2.238	5
	0.05	2.343	5
	0.10	2.132	50
0.01 M CBS/0.01 M CBS containing 0.2% gelatin	0.02	2.236	5
	0.05	2.158	5
	0.10	1.843	50

**Figure 2:**
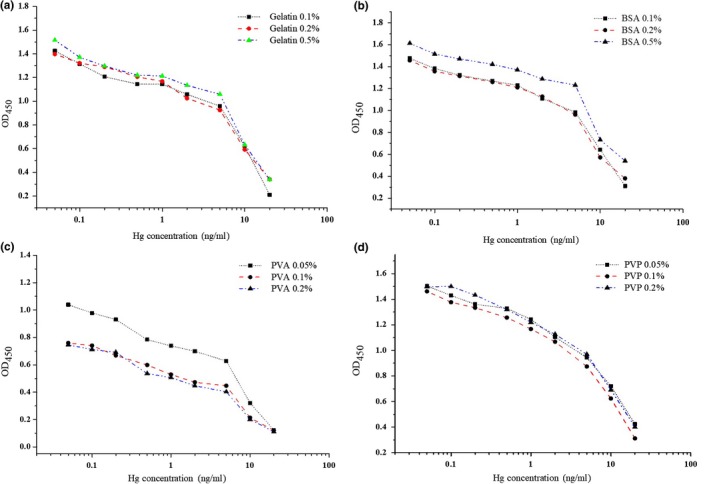
ELISA standard curves using CBS blocking solution with gelatin (0.1%, 0.2%, and 0.5%), BSA (0.1%, 0.2%, and 0.5%), PVA (0.05%, 0.1%, and 0.2%), and PVP (0.05%, 0.1%, and 0.2%)

Microwell plates treated with blocking solution containing PVA and BSA showed a positive stabilizing effect. Here, gelatin, BSA, PVA, and PVP were prepared and tested separately in the blocking solution and the antibody dilution buffer to assess their effect to Hg(II) detection. As shown in Figure 2, As shown in Figure 2, 0.5% gelatin and BSA in the blocking solution decreased the sensitivity for Hg(II) detection, which was consistent with other reports and previous results. As the high viscosity of PVA and PVP solutions, their maximum use concentration was 0.2%. In Figure 2c, ELISA containing PVA‐based blocking solution shows some color development inhibition resulting in decrease of OD value of blank, especially at 0.2% PVA condition. In Figure 2d, ELISA containing PVP‐based blocking solution shows better sensitivity than other three additives at trace Hg(II) existence condition. To sum up, blocking solution with 0.1% PVP was chosen as the optimum.

**Figure 3:**
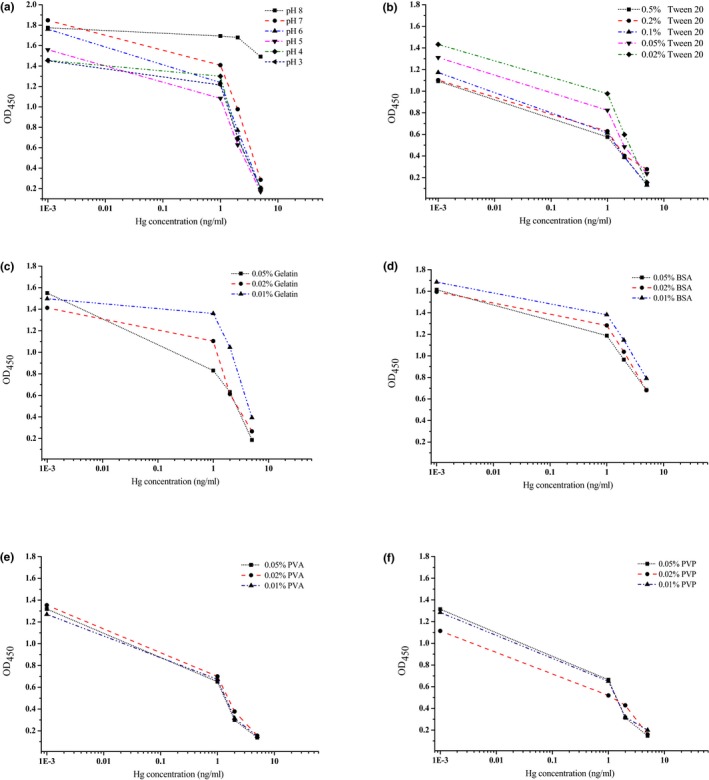
ELISA standard curves using Hg(II) dilution solution with pH (8, 7, 6, 5, 4, 3) and antibody dilution solution with Tween‐20 (0.5%, 0.2%, 0.1%, 0.05%, and 0.02%), gelatin (0.05%, 0.02%, and 0.01%), BSA (0.05%, 0.02%, and 0.01%), PVA (0.05%, 0.02%, and 0.01%), and PVP (0.05%, 0.02%, and 0.01%) in PBS buffer

The influence of varying the pH of the standard solution buffer to assay sensitivity was shown in Figure 3a. Hg(II) inhibition curves (1, 2, and 5 ng/ml Hg(II) were used here) were obtained at pH 3–8, and pH effects were evaluated mainly based on the sensitivity and protein stability. The absorbance values were depressed at pH 3 and pH 4. The sensitivity was decreased when pH was more than 7. The results indicating the assay have higher control OD values and are more sensitive under weak acid conditions, and Hg(II) dilution solution with pH 6 was used for the subsequent assays.

The influence of surfactant in antibody dilution buffer was shown in Figure 3b. Tween‐20 was the most commonly used reagent for reducing unspecific binding. At the condition of 0.05% Tween‐20 in PBS as the antibody dilution solution, the assay has higher control OD values and better sensitivity. Gelatin, BSA, PVA, and PVP were also prepared and tested separately in antibody dilution buffer to assess their effect to Hg(II) detection, and the results were shown in Figure 3c–f. Compared with PVP and PVA added, the addition of different concentration of gelatin or BSA in antibody dilution buffer caused obvious sensitivity decrease.

### 3.4 Characteristics of the optimized assay

**Figure 4:**
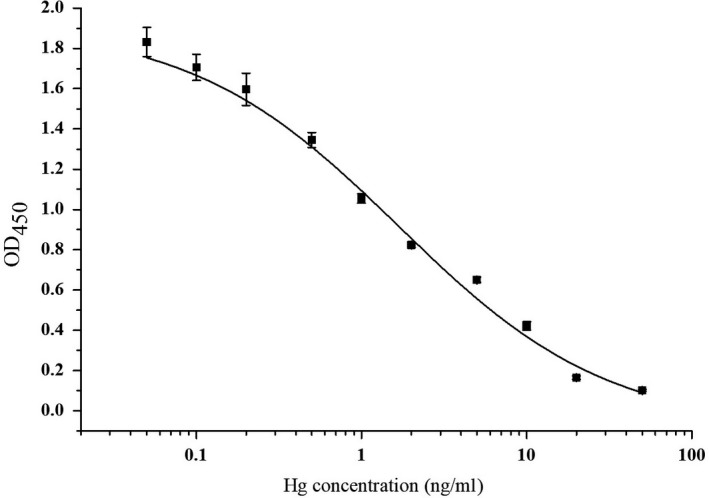
ELISA standard inhibition curve for analysis of Hg(II) under the optimal conditions

The optimized Hg(II) ELISA parameters were listed as follows: 1 μg/ml of Hg‐MNA‐OVA, CBS blocking solution with 0.1% PVP, Hg(II) standard solution prepared with 0.01 M PBS pH 6, 0.3 μg/ml of antibody concentration, 0.01 M PBS antibody dilution solution with 0.02% PVA, and 0.05% Tween‐20. As shown in Figure 4, the IC_50_ of the optimized immunoassay was 1.68 ng/ml with detection range (IC_20_–IC_80_) of 0.22–12.67 ng/ml. The limit of detection (LOD) was 0.079 ng/ml.

### 3.5 Cross‐reactivities (CRs) of antibody

The newly screened antibody A24 was highly sensitive and selective for the target analyte Hg(II), and the IC_50_ for CH_3_Hg was 12.8 ng/ml. Other metals, including Cr(III), Cr(VI), Mg(II), Cu(II), Ni(II), Fe(II), Co(II), Mn(II), Zn(II), Au(II), As(III), Ca(III), and Cd(II), were tested for CRs. The developed ELISA had low CRs for Au(II) (3.36%)，Cu(II) (0.84%), and negligible CRs for other tested metals (<0.84%). Therefore, the interference was negligible in tap and lake water detection.

### 3.6 Detection of Hg(II) in tap water and lake water by ELISA and ICP‐MS

To investigate the accuracy of the ELISA, tap water and lake water spiked with 1, 2, and 5 ng/ml Hg(II) were detected by immunoassay and ICP‐MS (Sun et al., 2018). A recovery of 80%–120% was acceptable for immunoassay. The tap water and lake water have found no Hg(II) existed by ICP‐MS. Recovery rates from ELISA ranged between 91% and 116%, whereas those obtained from the same samples by ICP‐MS were from 100.4% to 115.5% as shown in Table 4. The variable coefficients (CV) were from 5.6% to 16.6% for ELISA. Other relevant works also have similar recoveries (Wang et al., 2012; Zou et al., 2017). The accurate recovery of the spiked experiment suggests this method is a simple and suitable screening tool for the detection of Hg(II) water pollution.

**Table 4:** Recovery of Hg(II) spiked in tap water and lake water samples

Spiked concentration (ng/ml)	Detected by ELISA (mean, *n* = 3)		Detected by ICP‐MS (mean, *n* = 3)	
	Tap water	Lake water	Tap water	Lake water
1	1.12	1.09	1.11	1.14
2	2.32	1.95	2.03	2.31
5	4.72	4.55	5.33	5.02

## 4 CONCLUSION

Taken together, a direct competitive ELISA for the detection of Hg(II) in tap water and lake water samples has been developed and optimized. Hg(II) could be detected without chelation compared with our previous work. ELISA kit components were optimized for better sensitivity and decreasing interference. The IC_50_ for the developed assay was 1.68 ng/ml. The average recovery rates were between 91% and 116%. In our group, we have focused on application of heavy metal immunoassay for the detection of Pb(II) and Cd(II) in rice and wheat (Xu et al., 2016). Hg(II) pollution is rare in crops, and we will pay attention to Hg(II) in fish and kelp. The developed assay showed the antibody owned the potential for the detection of Hg(II) and CH_3_Hg in foods in future.

## CONFLICT OF INTEREST

The authors have declared that no conflict of interest exists.

## ETHICAL STATEMENT

All animal care and experimental protocols were ethically reviewed and approved by the Ethics Committee of Nanjing Agricultural University.
